# Antiangiogenic therapies in ovarian cancer

**DOI:** 10.1007/s12254-016-0282-4

**Published:** 2016-09-15

**Authors:** Alexander Reinthaller

**Affiliations:** Gynecologic Cancer Unit, Comprehensive Cancer Center Vienna, Department of Gynecology & Gynecologic Oncology, Medical University Vienna, Vienna, Österreich

**Keywords:** Antiangiogenic therapies, Advanced ovarian cancer, Recurrent ovarian cancer

## Abstract

Angiogenesis plays a pivotal role in normal ovarian physiology as well as in the formation and progression of ovarian cancer. Several well-designed phase II and III trials studied the efficacy of antiangiogenic agents in advanced ovarian cancer. The results of these trials demonstrated significantly prolonged progression-free survival when antiangiogenic agents were used as a maintenance therapy. To date, no effect on overall survival could be ascertained. The most widely studied antiangiogenic agent, bevacizumab – a monoclonal humanized antibody against vascular endothelial growth factor – was effective in all phases of the disease (first-line therapy, platinum-sensitive and platinum-resistant recurrence). These results led to regulatory approval in many countries including the European Union. Other anti-VEGF agents such as tyrosine kinase inhibitors have not shown increased activity but increased toxicity relative to bevacizumab. Agents targeting angiopoietin-1 and -2 are in development and new combinations with PARP inhibitors and immune checkpoint inhibitors are studied. This review summarizes the current data and knowledge on the clinical use of antiangiogenic agents in advanced ovarian cancer.

## Introduction

Epithelial ovarian cancer (EOC) is the leading cause of death from a gynecologic cancer. Currently there is no effective screening or early detection for this tumor entity. Therefore, 70–80 % of patients present at an advanced stage at the time of diagnosis. Standard therapy of advanced stage EOC is aggressive cytoreductive surgery with the aim of removing all visible tumor followed by platinum- and taxane-containing chemotherapy. With modern surgical interventions and contemporary chemotherapy, most patients attain complete clinical remission [1]. The majority of them, however, will eventually relapse and die of the disease. Therefore, additional therapeutic strategies are needed to improve patient survival. Angiogenesis has been established as a basic feature of tumor development, growth, and spread beyond regional borders [2]. Vascular endothelial growth factor (VEGF), which can be seen as the most prominent proangiogenic molecule, has been shown to be an independent prognostic parameter in patients with all stages of ovarian cancer [3]. Among several antiangiogenic compounds that have been developed, EOC proved to be a tumor entity particularly sensitive to antiangiogenic therapy. This review summarizes results of clinical trials investigating the efficacy and role of antiangiogenic therapies in advanced-stage EOC.

## Antiangiogenic agents in the adjuvant therapy of EOC

### Bevacizumab

Bevacizumab (BEV) is a monoclonal humanized antibody against VEGF. There are two randomized placebo-controlled phase III trials that investigated the efficacy of BEV in the adjuvant treatment of advanced EOC [4, 5]. GOG (Gynecologic Oncology Group) 218 was a double-blind, placebo-controlled, phase III trial that randomized patients with newly diagnosed stage III (incompletely resectable) or stage IV EOC who had undergone debulking surgery. Patients were randomized into three treatment groups: chemotherapy with paclitaxel plus carboplatin, for cycles 1–6 plus placebo in cycles 2–22 (control arm); chemotherapy plus BEV in cycles 2–6 and placebo in cycles 7–22 (BEV initiation treatment arm); and chemotherapy plus BEV in cycles 2–22 (BEV maintenance treatment arm). BEV was administered at a dosage of 15 mg/kg body weight. The primary endpoint of the study was progression-free survival (PFS). In all, 1873 women were enrolled in the study. The median PFS was 10.3 months in the control arm, 11.2 months in the BEV initiation arm, and 14.1 months in the BEV maintenance arm. Relative to control treatment, the hazard ratio for progression or death was 0.908 (*p* = 0.16) with BEV initiation and 0.717 (*p <* 0.001) with BEV maintenance. There were no significant differences in overall survival among the three groups. The rate of hypertension requiring medical therapy was higher in the BEV initiation group (16.5 %) and the BEV maintenance group (22.9 %) than in the control group (7.2 %). Gastrointestinal wall disruption requiring medical intervention occurred in 1.2 %, 2.8 %, and 2.6 % of patients in the control group, the BEV initiation group, and the BEV maintenance group, respectively [4].

In ICON, seven women with EOC were randomly assigned to carboplatin and paclitaxel given every 3 weeks for six cycles (control arm) and chemotherapy plus BEV (7.5 mg/kg body weight) given concurrently every 3 weeks for five or six cycles and continued for 12 additional cycles or until progression of disease. Outcome measures were PFS and overall survival. A total of 1528 women were randomly assigned to one of the two treatment regimens: 9 % had high-risk early-stage disease, 30 % were at high risk for progression, and 70 % had stage IIIC or IV EOC. PFS was 20.3 months with standard therapy compared with 21.8 months, measured at 36 months, with standard therapy plus BEV (*p* = 0.004). BEV was associated with increased toxicity (mainly hypertension; 18 % vs. 2 % with chemotherapy alone). In the updated analyses, PFS measured at 42 months was 22.4 months without BEV versus 24.1 months with BEV (*P* = 0.04); in patients at high risk for progression, the benefit was greater with BEV than without it, with PFS at 42 months of 14.5 months with standard therapy alone and 18.1 months with BEV added; the respective median overall survival was 28.8 and 36.6 months, respectively. BEV used as a maintenance therapy improved PFS in women with EOC. The benefits with respect to PFS and overall survival seemed to be greater among patients at high risk for disease progression and more advanced disease. In an exploratory analysis of a predefined subgroup of 502 patients with poor prognosis, a significant difference in overall survival was noted between women who received BEV plus chemotherapy and those who received chemotherapy alone (restricted mean survival time 34.5 months with standard chemotherapy vs. 39.3 months with BEV; *p =* 0·03) [5]. 

### Pazopanib

Pazopanib is an oral, tyrosine kinase inhibitor (TKI) of vascular endothelial growth factor receptor (VEGFR)-1/-2/-3, platelet-derived growth factor receptor (PDGFR), and c‑Kit. The AGO(Arbeitsgemeinschaft Gynäkologische Onkologie)-OVAR 16 study evaluated the role of pazopanib maintenance therapy in patients with EOC whose disease did not progress during first-line chemotherapy. In this study, 940 patients with histologically confirmed cancer of the ovary, fallopian tube, or peritoneum, FIGO stages II–IV, no evidence of progression after primary therapy consisting of surgery and at least five cycles of platinum-taxane chemotherapy were randomized 1:1 to receive pazopanib 800 mg once per day or placebo for up to 24 months. The primary endpoint was PFS. Maintenance pazopanib prolonged PFS compared with placebo (median 17.9 vs. 12.3 months, *p =* 0.0021). Overall survival did not show any significant difference. Grade 3 or 4 adverse events included hypertension (30.8 %), neutropenia (9.9 %), liver-related toxicity (9.4 %), diarrhea (8.2 %), fatigue (2.7 %), thrombocytopenia (2.5 %), and palmar-plantar erythrodysesthesia (1.9 %) and were significantly higher in the pazopanib arm. Pazopanib maintenance therapy significantly improved PFS in patients with advanced EOC [6, 7].

### Nintedanib

Nintedanib is an oral triple angiokinase inhibitor of VEGFR, PDGFR, and fibroblast growth factor receptor. In a double-blind phase III trial, chemotherapy-naive patients with FIGO stage IIB–IV EOC and upfront debulking surgery were stratified according to postoperative resection status, FIGO stage, and planned carboplatin dose. Patients were randomly assigned (2:1) to receive six cycles of carboplatin and paclitaxel in addition to either 200 mg of nintedanib (nintedanib group) or placebo (placebo group) twice daily on days 2–21 of every 3‑week cycle for up to 120 weeks. The primary endpoint was investigator-assessed PFS analyzed in the intention-to-treat population. In all, 1503 patients were screened and 1366 randomized: 486 (53 %) of 911 patients in the nintedanib group experienced disease progression or death compared with 266 (58 %) of 455 in the placebo group. Median PFS was significantly longer in the nintedanib group than in the placebo group (17.2 months vs. 16.6 months, *p* = 0.024). The most common adverse events were gastrointestinal (diarrhea grade 3/4: nintedanib group 22 % vs. placebo group 2 %) and hematological (neutropenia grade 3/4: nintedanib group 42 % vs. placebo group 36 %; thrombocytopenia: 18 % vs. 7 %; anemia: 13 % vs. 7 %, respectively). Nintedanib in combination with carboplatin and paclitaxel significantly increased PFS for women with advanced EOC, but is associated with more gastrointestinal adverse events [8].

## Recurrent EOC

### Platinum-sensitive EOC

In a randomized, blinded, placebo-controlled phase III trial the efficacy and safety of BEV with gemcitabine and carboplatin compared with gemcitabine and carboplatin in platinum-sensitive recurrent EOC was studied (OCEANS trial). BEV-naive patients with platinum-sensitive ovarian cancer (recurrence free interval >6 months after front-line platinum-based chemotherapy) and measurable disease were randomly assigned to gemcitabine/carboplatin plus either BEV or placebo for six to ten cycles. BEV was administered at a dosage of 15 mg/kg body weight. BEV or placebo was then continued until disease progression. The primary endpoint was PFS by RECIST (Response Evaluation Criteria In Solid Tumors). Secondary endpoints were objective response rate, duration of response, overall survival, and safety. In total, 484 patients were randomized. PFS for the BEV arm was superior to the placebo arm (median PFS was 12.4 vs. 8.4 months, *p >* 0.0001). The objective response rate (78.5 % vs. 57.4 %; *p <* 0.0001) and the duration of response (10.4 vs. 7.4 months) were significantly higher with the addition of BEV. No new safety concerns were noted. Gemcitabine/carboplatin plus BEV followed by BEV until progression prolonged PFS significantly compared with the placebo arm [9].

Cediranib is an oral antiangiogenic VEGFR 1–3 inhibitor. In ICON 6 – a randomized, three-arm, double-blind, placebo-controlled phase III trial – the efficacy and safety of cediranib in combination with platinum-based chemotherapy and as maintenance treatment in patients with platinum-sensitive recurrent EOC were assessed. Patients received up to six cycles of platinum-based chemotherapy and then entered a maintenance phase. Participants were randomly allocated (2:3:3) to receive (a) placebo with chemotherapy and as maintenance (control arm), (b) cediranib 20 mg once daily with chemotherapy then placebo as maintenance (concurrent arm), or (c) cediranib 20 mg once daily with chemotherapy and as maintenance (maintenance arm). Patients continued treatment to progression or unacceptable toxicity. The primary endpoint was PFS. Here, 456 patients were randomized and evaluated. Median PFS was 11.0 months in the maintenance arm, 8.7 months in the control arm (*p* < 0.0001), and 9.9 months in the concurrent arm. Diarrhea, neutropenia, hypertension, hypothyroidism, and voice changes were significantly more common in the cediranib arms. Of the patients, 10 % experienced grade 3/4 diarrhea during chemotherapy and cediranib therapy and 12 % during cediranib maintenance therapy. Cediranib showed a significantly prolonged PFS in the maintenance arm compared with the control arm, but was associated with significantly increased toxicity [10].

Trebananib inhibits the binding of angiopoietins 1 and 2 to the Tie2 receptor, and thereby inhibits angiogenesis. TRINOVA 1, a randomized, double-blind phase III study, assessed whether the addition of trebananib to single-agent weekly paclitaxel in patients with recurrent EOC improved PFS. Patients received weekly intravenous paclitaxel plus either weekly placebo or trebananib (15 mg/kg). Patients were stratified on the basis of platinum-free interval. The primary endpoint was PFS, and 919 patients were enrolled. Median progression-free survival was significantly longer in the trebananib group than in the placebo group (7.2 vs. 5.4 months, *p* < 0.0001). Trebananib was associated with more adverse event-related treatment discontinuations than was placebo and with higher incidences of chronic, long-lasting edema. Serious adverse events were recorded in 28 % vs. 34 % of patients in the trebananib group. Inhibition of angiopoietins 1 and 2 with trebananib provided a significant prolongation of PFS [11].

### Platinum-resistant EOC

AURELIA was a randomized phase III trial combining BEV with chemotherapy in platinum-resistant ovarian cancer. Eligible patients had measurable disease that had progressed less than 6 months after completing platinum-based therapy. Chemotherapy (pegylated liposomal doxorubicin, weekly paclitaxel, or topotecan) was chosen by the investigators. Patients were randomized to single-agent chemotherapy alone or in combination with BEV (10 mg/kg every 2 weeks or 15 mg/kg every 3 weeks) until progression and/or unacceptable toxicity. Single-agent BEV was permitted after progression with chemotherapy alone. The primary endpoint was PFS. Median PFS was 3.4 months with chemotherapy alone versus 6.7 months with BEV-containing therapy. Overall response rate (ORR) was 11.8 % versus 27.3 % (*p <* 0.001). No new safety signals were observed. Adding BEV to chemotherapy statistically significantly improved PFS and ORR [12].

## Discussion

Antiangiogenic agents clearly showed efficacy in patients with advanced EOC in a large number of well-designed phase III trials. BEV was the most intensively studied antiangiogenic agent. Adding BEV to chemotherapy and as a maintenance therapy led to a significantly improved PFS in first-line treatment as well as in platinum-sensitive and platinum-resistant EOC. During and after adjuvant therapy, an association has been shown between increasing disease severity and a stronger beneficial effect of BEV. These observations suggest that a residual tumor burden, presumably producing VEGF, is necessary to enable BEV to exert its effect on the tumor microenvironment. In patients with optimally cytoreduced advanced ovarian cancer, a later onset of BEV therapy and/or an extended duration might therefore be more effective.

TKIs also showed efficacy in the treatment of ovarian cancer in terms of PFS and response rates. However, compared with BEV, TKIs have a less favorable toxicity profile and therefore will probably lead to an impaired patient adherence to these orally administered drugs. Currently, none of the TKIs is approved in the European Union.

Several issues are still to be addressed regarding antiangiogenic therapy in EOC. The optimal duration of therapy remains unclear. It seems that prolonged administration of BEV (2 years) could improve PFS. Currently there is one randomized phase III trial investigating this issue (BOOST trial).

Another topic is re-induction therapy after complete response with an antiangiogenic agent.

Re-treatment with BEV after a prior BEV response was associated with a significantly improved PFS [13]. The meaningful improvement (14 months) in PFS strongly supports the re-use of BEV in subsequent regimens in patients who demonstrate an initial response to BEV. To clarify this issue, further clinical trials are needed.

Furthermore, the evaluation of biomarkers predicting the response to antiangiogenic therapies should be investigated. There is clear molecular evidence that ovarian cancer is a highly heterogeneous disease. The five main immunohistological subtypes (high-grade serous, endometrioid, clear cell, low-grade serous, and mucinous) differ vastly in terms of stage, chemosensitivity, overall survival, and driver genetic mutations [14]. Despite these clear differences, most clinical trials of antiangiogenesis therapy have been performed with unselected patient populations. One study as part of the ICON 7 trial demonstrated a discriminatory signature comprising mesothelin, FLT4, AGP, and CA-125 as potentially identifying patients with EOC more likely to benefit from BEV. The results require validation [15]. Molecular clustering showed different subtypes of ovarian cancers (immune, angio, angioimmune). Immune-type cancers seem to be very sensitive to platinum chemotherapy and the addition of BEV showed a negative impact on clinical outcome [16].

In summary, improved patient selection and identification of patients who will benefit from antiangiogenic therapy and those who will not are therefore of utmost importance.
